# Accumulation of Epstein-Barr virus–induced cross-reactive immune responses is associated with multiple sclerosis

**DOI:** 10.1172/JCI184481

**Published:** 2024-11-01

**Authors:** Hannes Vietzen, Laura M. Kühner, Sarah M. Berger, Philippe L. Furlano, Gabriel Bsteh, Thomas Berger, Paulus Rommer, Elisabeth Puchhammer-Stöckl

**Affiliations:** 1 Center for Virology,; 2 Department of Neurology, and; 3 Comprehensive Center for Clinical Neurosciences and Mental Health, Medical University of Vienna, Vienna, Austria.

**Keywords:** Autoimmunity, Virology, Autoimmune diseases, Multiple sclerosis

## To the Editor:

Multiple sclerosis (MS) is a chronic autoinflammatory and demyelinating disease of the central nervous system (CNS). The individual risk of developing MS substantially increases after a primary infection with the ubiquitous EBV (1). MS pathogenesis was associated earlier with the development of high-level antibodies directed against the EBV nuclear antigen-1 (EBNA-1), which cross-reacts with distinct CNS-derived proteins and may result in autoreactive immune responses (1). Recent studies have already identified a conserved sequence in the EBNA-1 region, EBNA381–452, as a main factor for potential molecular mimicry and autoreactivity (1). However, it is unknown whether potent EBNA381–452–specific immune responses may in fact trigger MS pathogenesis in the individual host.

The aim of this study was thus to investigate whether a distinct antibody signature against EBNA381–452 is associated with high cross-reactive autoimmune responses that further evolve into MS. Therefore, we recruited a study cohort of 270 EBV-seropositive MS patients that were matched to 270 healthy controls regarding age, sex, and time point of EBV seroconversion (Supplemental Table 1; supplemental material available online with this article; https://doi.org/10.1172/JCI184481DS1). From all study participants, plasma samples were available at the time point of primary EBV infection as well as at MS diagnosis and, in controls, at a matched time point after EBV seroconversion, respectively.

**Figure 1:**
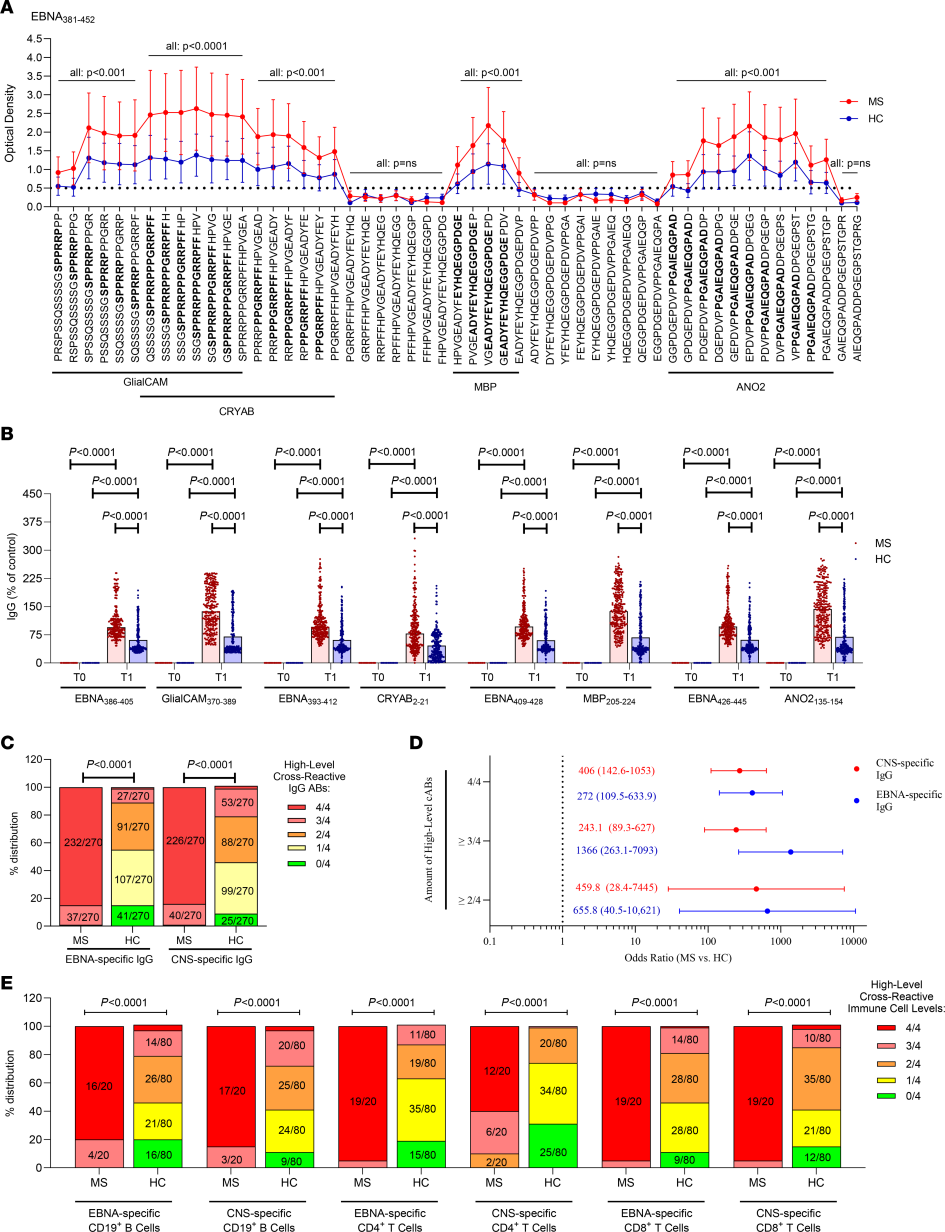
Accumulation of EBNA_381–452_–mediated immune responses is associated with MS. (**A**–**D**) EBNA-specific IgG antibody responses were assessed in *n* = 270 MS patients and *n* = 270 healthy controls using indicated (**A**) EBNA_381–452_–derived peptides or (**B**) individual EBNA_386–405_–,EBNA_393–412_–,EBNA_409–428_–,EBNA_426–445_–, or corresponding CNS-derived GlialCAM_370–389_–,CRYAB_2–21_–,MBP_205–224_-,or ANO2_135–154_ peptides. IgG antibody responses were assessed at the time point of MS diagnosis or a matched time point for controls (T1; **A** and **B**) and during primary EBV infection (T0; **A**). The dashed black line indicates the cut-off for detection. (**C** and **D**) High-level EBNA-specific or CNS-specific antibody levels were first defined by receiver operating characteristic (ROC) analysis (Supplemental Table 2) and then compared between MS patients and controls. (**E**) Distribution of high-level EBNA-specific or CNS-specific CD19^+^ B cell (Figure 1B), CD4^+^ T cell (**C**), and CD8^+^ T cell (**D**) levels was defined by ROC analysis (Supplemental Table 2) and then compared between *n* = 20 MS patients and *n* = 80 healthy controls. Statistical differences were assessed by (**A**) Kruskal-Wallis and Dunn’s multiple-comparison test, (**B**) Mann-Whitney *U* test, (**C**–**E**) and χ^2^ test. ABs, antibodies; HC, healthy control; T, timepoint.

We first analyzed the fine specificity of the EBNA381–452–specific antibody responses of all MS patients and controls using an EBNA381–452–derived peptide library. MS patients had during MS diagnosis, in comparison with controls at a matched time point, significantly higher EBNA-specific antibody responses against epitopes that exhibit recently identified cross-reactive immune responses to the CNS-derived glial cell adhesion molecule (GlialCAM) (2), α-crystallin B chain (CRYAB) (3), myelin basic protein (MBP) (4), and anoctamin 2 (ANO2) (5) (Figure 1A).

Hence, we further quantified the individual antibody responses against all four EBNA-derived and all four associated cross-reactive CNS-derived peptides (EBNA386–405/GlialCAM370–389, EBNA393–412/CRYAB2–21, EBNA409–428/MBP205–224, EBNA426–445/ANO2135–154) in the individual MS patients and controls. Neither MS patients nor controls had detectable EBNA381–452–specific antibody responses at the time point of primary EBV infection (Figure 1B).

At the time point of MS diagnosis, overall higher EBNA- and CNS-derived peptide-specific antibody titers were observed in MS patients than in controls (Figure 1B). However, a substantial part (33.8%–53%) of controls had peptide-specific antibody titers against individual EBNA- and CNS-derived peptides that were comparable to titers found in MS patients. Neither in MS patients nor in healthy controls were a particular combination of EBNA- or CNS-derived peptide-specific antibody titers significantly overrepresented. We therefore hypothesized that mainly the accumulation of high titers of cross-reactive EBNA- and CNS-peptide-specific antibody responses is associated with the individual development of MS. Hence, we first calculated individual EBNA- and CNS-derived peptide-specific IgG antibody titers that are associated with an increased risk for MS, defining each peptide-specific antibody titer above the individual cut-off as an independent risk factor (Supplemental Table 2). Next, we compared the number of independent high-level antibody titers between MS patients and controls. As shown in Figure 1C, 99.6% and 98.5% of MS patients, but only 11.5% and 21.5% of controls, had high-level EBNA- and CNS-derived antibody titers against three or more of the cross-reactive regions. The presence of high-level EBNA-specific antibody responses against three or more cross-reactive peptide regions was associated with a 1,366-fold increased risk for the development of MS (Figure 1D).

To investigate whether MS patients are also hallmarked by an accumulation of high-level EBNA- and CNS-derived peptide-specific B cell and T cell responses, we then collected PBMCs of n = 20 MS patients and n = 80 healthy controls and quantified all EBNA- and CNS-specific CD19+ B cell, CD4+ T cell, and CD8+ T cell levels by flow cytometry. We found an overall high correlation between EBNA- and CNS-derived peptide-specific cell levels and respective antibody responses (all: r2 < 0.61,Supplemental Figure 1A). Going along with this finding, MS patients showed significantly higher EBNA-derived and CNS-derived immune cell levels than healthy controls (Supplemental Figure 1, B–D).

We then calculated individual EBNA-derived and CNS-derived peptide-specific CD19+ B cell, CD4+ T cell, and CD8+ T cell levels that, independently of the EBNA- and CNS-derived peptide-specific antibody titers, were associated with an increased risk for MS (Supplemental Table 3). We then compared the number of high-level B cell and T cell responses between MS patients and healthy controls. MS patients frequently showed an accumulation of high-level CD19+ B cell, CD4+ T cell, and CD8+ T cell responses directed against all four EBNA- or CNS-derived peptides, while this was rare in the control populations (Figure 1E).

In summary, we report that MS patients are hallmarked by an accumulation of immune responses specifically directed against EBV EBNA381–452 and cross-reacting with CNS GlialCAM370–389–, CRYAB2–21–, MBP205–224–, and ANO2135–154–derived epitopes. Although, our findings provide additional insights into the pathogenesis of MS, further longitudinal studies are required to assess the individual increase of EBNA381–452–derived antibody responses.

## Supplementary Material

Supplemental data

Supporting data values

## Version 1 11/01/2024

Electronic publication
