# Supplementary material for: Accumulation of Epstein-Barr virus–induced cross-reactive immune responses is associated with multiple sclerosis
Source: J Clin Invest. 2024 Nov 1;134(21):e184481. doi: 10.1172/JCI184481 (PMC11527435; doi:10.1172/JCI184481)
Supplement: Supplemental data [file jci-134-184481-s110.pdf]

Supplementary Information

Table of Content

Supplementary Information 2

Sex as a Biological Variable 2

Data Availability Statement 2

Ethics Declaration 2

Study Cohort 2

Peptides 5

Detection of Peptide-specific IgG Antibodies 5

Identification of Peptide-specific Cells 5

Statistical analysis 6

Supplementary References 6

Supplementary Tables 7

Table S1: Characteristics of the Study Cohort 7

Table S2: Peptide Sequences 7

Table S3: ROC analysis to define high-level EBNA- and CNS-peptide-specific immune responses 10

Supplementary Figures 13

Figure S1 13

Supplementary Information

Sex as a Biological Variable

Our study examined male and female MS patients and healthy controls. MS patients were matched to healthy controls according to the sex of the study participants. Similar findings are reported for both sexes.

Data Availability Statement

De-identified data are available from the corresponding author (hannes.vietzen@meduniwien.ac.at) upon request and upon approval by the data-clearing committee of the Medical University of Vienna.

Ethics Declaration

The study was approved by the Institutional Review Board of the Medical University of Vienna (IRB number: 1339/2022).

Study Cohort

For this single-center case-control study, a total of N=270 EBNA-1-seropositive MS patients and N=270 EBNA-1-seropositive healthy controls were included. The recruitment of the study participants was recently described in detail (1). In brief, a total of 12,708 EBV-seropositive MS patients and healthy controls were initially recruited. From all these individuals, N=270 MS patients fulfilled the following inclusion criteria:

1. MS diagnosis according to the McDonald criteria (2).

2. Retrospective follow-up serum samples (with maximal intervals of 12 months) until EBV seroconversion of the patients serologically proven by EBV-specific IgG antibodies.
3. EBV-EBNA-1-specific antibodies detectable at the time of MS diagnosis.
4. Clinical information about infectious mononucleosis diagnosis available.

All MS patients were part of the Vienna Multiple Sclerosis Database (VMSD). Within this database, plasma, serum and cerebrospinal fluid samples were collected upon consent at diagnosis and regularly during the course of the disease. The majority of MS patients were of European (N=252), followed by Middle Eastern (N=17) and Asian (N=1) nationality. None of the patients enrolled were treated with MS-specific treatment before diagnosis. 38 MS patients (14.1%) were treated with corticosteroids within two months prior to MS diagnosis. At the time of diagnosis, 24.4%, 23.7%, and 51.9% of MS patients had ≤3, 4-9, and ≥10 lesions on T2-weighted brain magnetic resonance tomography, respectively. For each patient with MS, one healthy control individual was included in the study. Control individuals were healthy, voluntary blood donors without a history of MS or other autoimmune diseases or neuro-inflammatory disorders. Controls were matched according to sex, age, time of EBV seroconversion, and the occurrence of infectious mononucleosis (IM) in the past to the MS cohort (Table S1). All controls fulfilled the following inclusion criteria:

1. No diagnosis of MS or other autoimmune disease or neuro-inflammatory disorder.
2. Retrospective follow-up serum samples (with maximal intervals of 12 months) until EBV seroconversion of the patients serologically proven by EBV-specific IgG antibodies at a matched time point for MS diagnosis.
3. EBV-EBNA-1-specific antibodies detectable at the time of MS diagnosis.
4. Clinical information about infectious mononucleosis diagnosis available.

From all study participants, retrospective follow-up serum samples in a 1-11 (median: 4.2) month interval until EBV seroconversion were available. N=202 (74.8%) MS patients and N=202 (74.8%) healthy controls had clinically evident infectious mononucleosis (IM) during EBV seroconversion. IM was clinically confirmed in all patients by the triad of fever, lymphadenopathy, and tonsillitis, and serologically by EBV-VCA-specific IgM, but non-detectable EBV-EBNA-specific IgG antibodies. The time of seroconversion in IM patients was defined as clinical evidence of IM together with the plasma sample with the first detectable EBV-VCA-specific IgM antibodies.

68 (25.2%) patients with MS had an asymptomatic primary EBV infection, hallmarked by the absence of any clinical symptoms during seroconversion. From all MS patients with asymptomatic primary EBV infection, retrospective follow-up serum samples in a 1-9 (median: 4.2) month interval until EBV seroconversion were available. The time of seroconversion in these patients was defined as the plasma sample with the first EBV-VCA-specific IgM and/or EBV-EBNA-specific IgG and/or EBV-VCA-specific IgG antibodies, following an EBV seronegative sample. The seroconversion occurred 1.4–4.5 (median: 3.1) months after the last EBV-seronegative sample. For all MS patients, the time between seroconversion and MS was defined as the time between the first time EBV-seropositive sample and the time of the first clinically proven MS diagnosis.

In this study, two plasma samples from each study participant were included: One plasma sample was collected from patients or controls during the primary EBV infection. One additional plasma sample was collected from patients immediately (0-6 days) after MS diagnosis or at a matched time point after EBV seroconversion for the controls.

From 20 MS patients (7.4%) and 80 controls (29.6%), additional peripheral blood mononuclear cells (PBMC) were available, which were collected from MS patients immediately (0-6 days) after MS diagnosis, or at a matched time point after EBV seroconversion for the controls. PBMCs from

MS patients and healthy controls were isolated from whole blood by Ficoll-Paque PLUS density (Cytiva) gradient centrifugation according to the manufacturer's instructions and subsequently stored frozen at −80 °C in $5x10^6$ viable cells per aliquot in 90% FCS + 10% DMSO (both Thermo-Fisher).

Peptides

All peptides (Table S2) were synthesized at 95% purity (Peptides&Elephants).

Detection of Peptide-specific IgG Antibodies

Peptide-specific IgG antibodies were quantified by ELISA as described before (1). Briefly, 96-well plates (Thermo Fisher Scientific) were coated with 1 μg/mL of respective peptides (Table S2) and then incubated with 1:100 diluted patient plasma. Peptide-specific IgG antibodies were detected using HRP-conjugated goat anti-human IgG (1:12500, Thermo Fisher Scientific, REF: #31412) and TMB substrate (both Thermo Fisher). The cut-off for detection was defined as the 95.0% CI as described before (3).

Identification of Peptide-specific Cells

Peptide-specific $CD4^+$ T cells, $CD8^+$ T cells, and $CD19^+$ B cells were identified by flow cytometry using respective peptides (Table S2) as described in detail before (1).

Statistical analysis

Contingency tables were assessed by the χ^2-Test test. Outliers of the antibody titers and cell levels were first identified using the ROUT method and then compared between the groups using Kruskal-Wallis and Dunn's multiple comparison test or the Mann-Whitney-Test. Statistical significance was set at $p < 0.05$. Statistical differences were assessed using GraphPad Prism 10.

Supplementary References

1. Vietzen H, Berger SM, Kühner LM, Furlano PL, Bsteh G, Berger T, et al. Ineffective control of Epstein-Barr-virus-induced autoimmunity increases the risk for multiple sclerosis. *Cell.* 2023;186(26):5705-18.e13.
2. Thompson AJ, Banwell BL, Barkhof F, Carroll WM, Coetzee T, Comi G, et al. Diagnosis of multiple sclerosis: 2017 revisions of the McDonald criteria. *Lancet Neurol.* 2018;17(2):162-73.
3. Frey A, Di Canzio J, and Zurakowski D. A statistically defined endpoint titer determination method for immunoassays. *Journal of immunological methods.* 1998;221(1-2):35-41.

Supplementary Tables

Table S1: Characteristics of the Study Cohort

Characteristic	MS<br>N=270	Healthy Control<br>N=270
**Sex**<br>Female<br>Male	 N=168 (62.2 %)<br>N=102 (37.8 %)	 N=168 (62.2 %)<br>N=102 (37.8 %)
**Median Age** (min-max)	37.2 (16.3 - 59.7)	34.7 (18.2 - 51.3)
**EBV-seroconversion** (median years) **pre-MS diagnosis or matched time point** for healthy controls (range)	8.2 (3.1 – 11.8)	7.7 (2.9 – 12.4)
**Primary EBV-Infection**<br>Infectious Mononucleosis<br>Asymptomatic	 N=202 (74.8 %)<br>N= 68 (25.1 %)	 N=202 (74.8 %)<br>N= 68 (25.1 %)
**EBV EBNA-1 IgG Titer** [DRG Units/mL] **at MS diagnosis or matched time point** for healthy controls	37.91<br>(±7.28)	33.28<br>(±9.7)
**Total IgG concentration** [mg/dL] **during primary EBV-infection** (range)	1203.4<br>(703.6 - 1693.6)	1167.1<br>(709.3 - 1698.2)
**Total IgG concentration** [mg/dL] **during MS diagnosis or matched time point** for healthy controls (range)	1317.7<br>(851.3 - 1847.3)	1197.5<br>(701.3 - 1686.1)

Table S2: Peptide Sequences

ID	Peptide Sequence
$EBNA_{381-452}$-derived peptide library	
Peptide_01	PRSPSSQSSSSGSPPRRPPP
Peptide_02	RSPSSQSSSSGSPPRRPPPG
Peptide_03	SPSSQSSSSGSPPRRPPPGR
Peptide_04	PSSQSSSSGSPPRRPPPGRR
Peptide_05	SSQSSSSGSPPRRPPPGRRP

Peptide_06	SQSSSSGSPPRRPPPGRRPF
Peptide_07	QSSSSGSPPRRPPPGRRPFF
Peptide_08	SSSSGSPPRRPPPGRRPFFH
Peptide_09	SSSGSPPRRPPPGRRPFFHP
Peptide_10	SSGSPPRRPPPGRRPFFHPV
Peptide_11	SGSPPRRPPPGRRPFFHPVG
Peptide_12	GSPPRRPPPGRRPFFHPVGE
Peptide_13	SPPRRPPPGRRPFFHPVGEA
Peptide_14	PPRRPPPGRRPFFHPVGEAD
Peptide_15	PRRPPPGRRPFFHPVGEADY
Peptide_16	RRPPPGRRPFFHPVGEADYF
Peptide_17	RPPPGRRPFFHPVGEADYFE
Peptide_18	PPPGRRPFFHPVGEADYFEY
Peptide_19	PPGRRPFFHPVGEADYFEYH
Peptide_20	PGRRPFFHPVGEADYFEYHQ
Peptide_21	GRRPFFHPVGEADYFEYHQE
Peptide_22	RRPFFHPVGEADYFEYHQEG
Peptide_23	RPFFHPVGEADYFEYHQEGG
Peptide_24	PFFHPVGEADYFEYHQEGGP
Peptide_25	FFHPVGEADYFEYHQEGGPD
Peptide_26	FHPVGEADYFEYHQEGGPDG
Peptide_27	HPVGEADYFEYHQEGGPDGE
Peptide_28	PVGEADYFEYHQEGGPDGEP

Peptide_29	VGEADYFEYHQEGGPDGEPD
Peptide_30	GEADYFEYHQEGGPDGEPDV
Peptide_31	EADYFEYHQEGGPDGEPDVP
Peptide_32	ADYFEYHQEGGPDGEPDVPP
Peptide_33	DYFEYHQEGGPDGEPDVPPG
Peptide_34	YFEYHQEGGPDGEPDVPPGA
Peptide_35	FEYHQEGGPDGEPDVPPGAI
Peptide_36	EYHQEGGPDGEPDVPPGAIE
Peptide_37	YHQEGGPDGEPDVPPGAIEQ
Peptide_38	HQEGGPDGEPDVPPGAIEQG
Peptide_39	QEGGPDGEPDVPPGAIEQGP
Peptide_40	EGGPDGEPDVPPGAIEQGPA
Peptide_41	GGPDGEPDVPPGAIEQGPAD
Peptide_42	GPDGEPDVPPGAIEQGPADD
Peptide_43	PDGEPDVPPGAIEQGPADDP
Peptide_44	DGEPDVPPGAIEQGPADDPG
Peptide_45	GEPDVPPGAIEQGPADDPGE
Peptide_46	EPDVPPGAIEQGPADDPGEG
Peptide_47	PDVPPGAIEQGPADDPGEGP
Peptide_48	DVPPGAIEQGPADDPGEGPS
Peptide_49	VPPGAIEQGPADDPGEGPST
Peptide_50	PPGAIEQGPADDPGEGPSTG
Peptide_51	PGAIEQGPADDPGEGPSTGP

Peptide_52	GAIEQGPADDPGEGPSTGPR
Peptide_53	AIEQGPADDPGEGPSTGPRG
EBNA-derived Peptides	
$EBNA_{386-405}$	SQSSSSGSPPRRPPPGRRPF
$EBNA_{393-412}$	SPPRRPPPGRRPFFHPVGEA
$EBNA_{409-428}$	VGEADYFEYHQEGGPDGEPD
$EBNA_{426-445}$	EPDVPPGAIEQGPADDPGEG
CNS-derived Peptides	
$GlialCAM_{370-389}$	ATGRTHSSPPRAPSSPGRSR
$CRYAB_{2-21}$	DIAIHHPWIRRPFFPFHSPS
$MBP_{205-224}$	GKGRGLSLSRFSWGAEQQRP
$ANO2_{135-154}$	PHAGGPGDIELGPLDALEEE

Table S3: ROC analysis to define high-level EBNA- and CNS-peptide-specific immune responses

Peptide-specific IgG	Cut-Off *	Area under the Curve	Sensitivity % (95% CI)	Specificity % (95% CI)
$EBNA_{386-405}$	< 59.50%	0.82	65.31% (59.47% to 70.73%)	97.41% (94.75% to 98.74%)
$GlialCAM_{370-389}$	< 46.74%	0.84	59.78% (53.84% to 65.44%)	100% (98.60% to 100.0%)
$EBNA_{393-412}$	< 61.97%	0.82	66.05% (60.22% to 71.43%)	93.7% (90.15% to 96.03%)
$CRYAB_{2-21}$	< 33.08%	0.75	46.86% (41.01% to 52.81%)	87.78% (83.33% to 91.16%)
$EBNA_{409-428}$	< 57.04%	0.82	63.84% (57.96% to 69.33%)	96.3% (93.32% to 97.98%)
$MBP_{205-224}$	< 52.99%	0.84	61.25% (55.34% to 66.86%)	97.41% (94.75% to 98.74%)
$EBNA_{426-445}$	< 51.15%	0.81	60.89%	98.15%

			(54.96% to 66.50%)	(95.74% to 99.21%)
ANO2$_{135-154}$	< 56.89%	0.84	63.1%<br>(57.21% to 68.62%)	97.04%<br>(94.26% to 98.49%)
**Peptide-specific CD19^{+} B cells**	**Cut-Off ****	**Area under the Curve**	**Sensitivity %<br>(95% CI)**	**Specificity %<br>(95% CI)**
EBNA$_{386-405}$	< 0.001583%	0.80	61.73%<br>(50.84% to 71.55%)	100%<br>(83.89% to 100.0%)
GlialCAM$_{370-389}$	< 0.001064%	0.77	60.49%<br>(49.61% to 70.43%)	100%<br>(98.89% to 100.0%)
EBNA$_{393-412}$	< 0.001854%	0.78	58.02%<br>(47.15% to 68.17%)	100%<br>(83.89% to 100.0%)
CRYAB$_{2-21}$	< 0.0003797%	0.70	39.51%<br>(29.57% to 50.39%)	95%<br>(76.39% to 99.74%)
EBNA$_{409-428}$	< 0.002244%	0.76	65.43%<br>(54.59% to 74.88%)	85%<br>(63.96% to 94.76%)
MBP$_{205-224}$	< 0.001806%	0.79	59.26%<br>(48.38% to 69.30%)	100%<br>(83.89% to 100.0%)
EBNA$_{426-445}$	< 0.001724%	0.74	53.09%<br>(42.33% to 63.57%)	95%<br>(76.39% to 99.74%)
ANO2$_{135-154}$	< 0.001815%	0.72	60.49%<br>(49.61% to 70.43%)	90%<br>(69.90% to 98.22%)
**Peptide-specific CD4^{+} T cells**	**Cut-Off****	**Area under the Curve**	**Sensitivity %<br>(95% CI)**	**Specificity %<br>(95% CI)**
EBNA$_{386-405}$	< 0.3250%	0.86	65.43%<br>(54.59% to 74.88%	95%<br>(76.39% to 99.74%)
GlialCAM$_{370-389}$	< 0.1340%	0.87	70.37%<br>(59.69% to 79.21%)	95%<br>(76.39% to 99.74%)
EBNA$_{393-412}$	< 0.2998%	0.73	46.91%<br>(36.43% to 57.67%)	100%<br>(83.89% to 100.0%)
CRYAB$_{2-21}$	< 0.1256%	0.86	72.84%<br>(62.28% to 81.33%)	95%<br>(76.39% to 99.74%)
EBNA$_{409-428}$	< 0.7113%	0.99	98.77%<br>(93.33% to 99.94%)	100%<br>(83.89% to 100.0%)
MBP$_{205-224}$	< 0.1223%	0.79	92.59%<br>(84.77% to 96.56%)	65%<br>(43.29% to 81.88%)
EBNA$_{426-445}$	< 0.1777%	0.82	53.09%<br>(42.33% to 63.57%)	100%<br>(83.89% to 100.0%)
ANO2$_{135-154}$	< 0.1165%	0.85	65.43%<br>(54.59% to 74.88%)	100%<br>(83.89% to 100.0%)
**Peptide-specific CD8^{+} T cells**	**Cut-Off****	**Area under the Curve**	**Sensitivity %<br>(95% CI)**	**Specificity %<br>(95% CI)**
EBNA$_{386-405}$	< 0.01128%	0.78	59.26%<br>(48.38% to 69.30%)	100%<br>(83.89% to 100.0%)
GlialCAM$_{370-389}$	< 0.004573%	0.79	59.26%<br>(48.38% to 69.30%)	100%<br>(83.89% to 100.0%)

$EBNA_{393-412}$	< 0.01133%	0.76	60.49% (49.61% to 70.43%)	95% (76.39% to 99.74%)
$CRYAB_{2-21}$	< 0.002575%	0.77	56.79% (45.94% to 67.03%)	100% (83.89% to 100.0%)
$EBNA_{409-428}$	< 0.008129%	0.79	59.26% (48.38% to 69.30%)	100% (83.89% to 100.0%)
$MBP_{205-224}$	< 0.002522%	0.79	55.56% (44.73% to 65.88%)	100% (83.89% to 100.0%)
$EBNA_{426-445}$	< 0.004754%	0.79	55.56% (44.73% to 65.88%)	100% (83.89% to 100.0%)
$ANO2_{135-154}$	< 0.002894%	0.79	64.2% (53.33% to 73.78%)	95% (76.39% to 99.74%)

* Antibody levels were compared between N=270 MS patients and N=270 healthy controls.

** $CD19^+$ B cell, $CD4^+$ T cell, and $CD8^+$ T cell frequencies were compared between N=20 MS patients and N=80 healthy controls.

Supplementary Figures

Figure S1

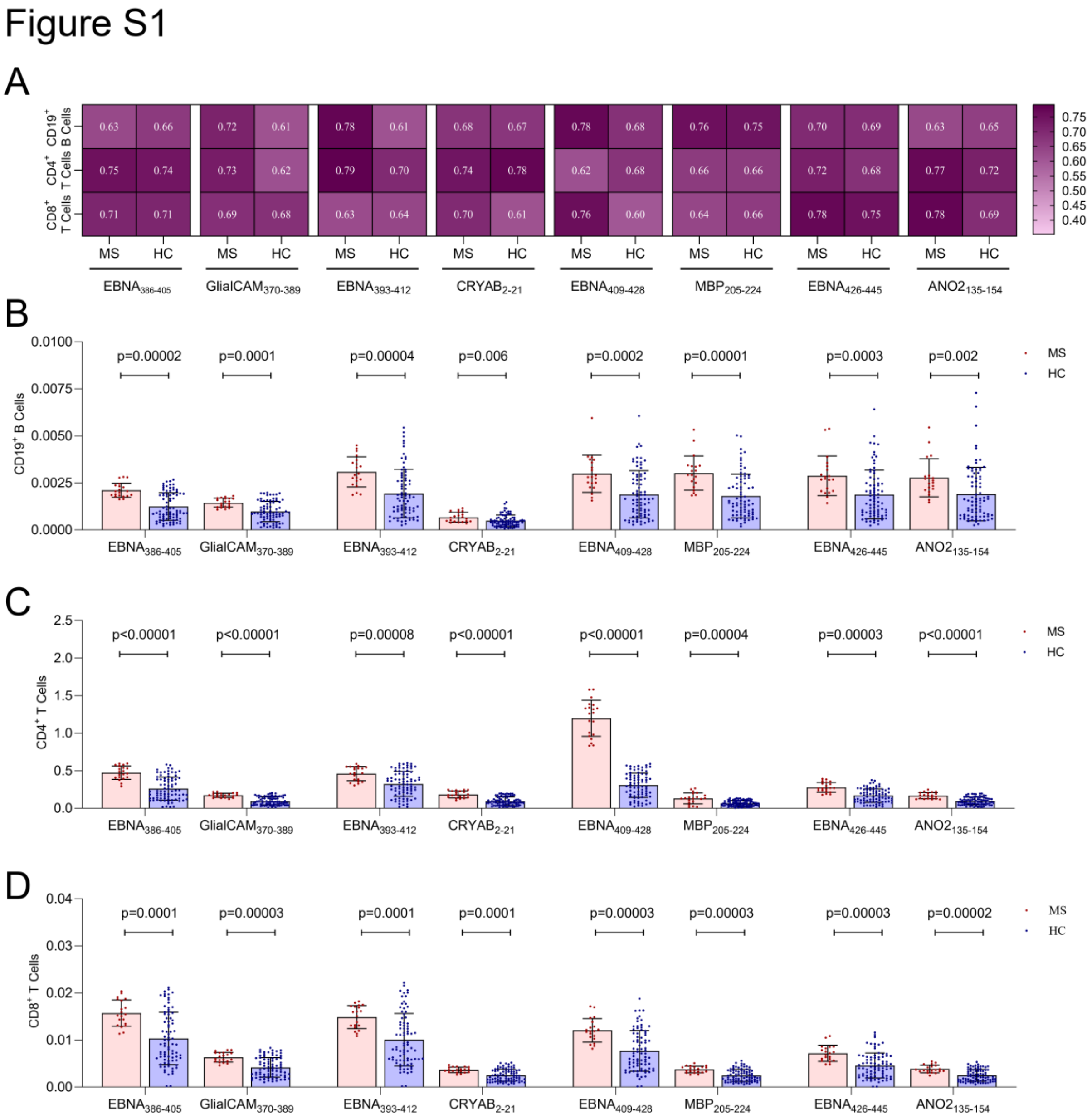

Figure S1: (A) EBNA-specific or CNS-specific $CD4^+$ T cells, CD8+ T cells, and $CD19^+$ B cell levels were assessed in N=20 MS patients and N=80 healthy controls. Heatmap shows the individual coefficients of determination (r2) from non-linear regression between EBNA-derived and CNS-derived antibody titers and EBNA-derived and CNS-derived $CD19^+$ B cell, $CD4^+$ T cell, and $CD8^+$ T cell levels. (B-D) $EBNA_{386-405}$-, $EBNA_{393-412}$-, $EBNA_{409-428}$-, $EBNA_{426-445}$- or corresponding CNS-derived $GlialCAM_{370-389}$-, $CRYAB_{2-21}$-, $MBP_{205-224}$-, $ANO2_{135-154}$- peptides-specific (B) $CD19^+$ B cell, (C) $CD4^+$ T cell, or (D) $CD8^+$ T cell levels were assessed in N=20 MS patients and N=80 healthy controls by flow-cytometry. (B-D) Statistical differences were assessed between the groups by Kruskal-Wallis and Dunn's multiple comparison test. **ABs**: Antibodies, **HC**: Healthy Control, **MS**: Multiple Sclerosis.
